# Shaping Ability of Single-file Systems with Different Movements: A Micro-computed Tomographic Study

**DOI:** 10.7508/iej.2016.03.016

**Published:** 2016-05-01

**Authors:** Joedy Santa-Rosa, Manoel Damião de Sousa-Neto, Marco Aurelio Versiani, Giselle Nevares, Felipe Xavier, Kaline Romeiro, Marcely Cassimiro, Graziela Bianchi Leoni, Rebeca Ferraz de Menezes, Diana Albuquerque

**Affiliations:** a *Department of Operative Dentistry and Endodontics, Dental College of Pernambuco, University of Pernambuco, Pernambuco, Brazil; *; b * Department of Restorative Dentistry, Faculty of Dentistry, University of São Paulo, Ribeirão Preto, Brazil*

**Keywords:** Micro-Computed Tomography, OneShape, Reciprocating Motion, Rotary System, WaveOne

## Abstract

**Introduction:**

This study aimed to perform a rigorous sample standardization and also evaluate the preparation of mesiobuccal (MB) root canals of maxillary molars with severe curvatures using two single-file engine-driven systems (WaveOne with reciprocating motion and OneShape with rotary movement), using micro-computed tomography (micro-CT).

**Methods and Materials:**

Ten MB roots with single canals were included, uniformly distributed into two groups (*n*=5). The samples were prepared with a WaveOne or OneShape files. The shaping ability and amount of canal transportation were assessed by a comparison of the pre- and post-instrumentation micro-CT scans. The Kolmogorov-Smirnov and t-tests were used for statistical analysis. The level of significance was set at 0.05.

**Results:**

Instrumentation of canals increased their surface area and volume. Canal transportation occurred in coronal, middle and apical thirds and no statistical difference was observed between the two systems (*P*>0.05). In apical third, significant differences were found between groups in canal roundness (in 3 mm level) and perimeter (in 3 and 4 mm levels) (*P*<0.05).

**Conclusion:**

The WaveOne and One Shape single-file systems were able to shape curved root canals, producing minor changes in the canal curvature.

## Introduction

Instrumentation of the root canals can lead to alterations in shape of the canal [1], transportation [2] and even perforation [3]. To remove the contaminated dentin and meanwhile shape the root canal, it is important to conform to the natural anatomy to minimize the damage to the tooth structure.

The use of single-file nickel-titanium (NiTi) engine-driven files in root canal preparation has increased and different systems have been developed [4, 5]. WaveOne (Dentsply, Tulsa Dental, Tulsa, OK, USA) is amoung these single-file systems which is used with a specific motor that performs reciprocating movements, *i.e.*, movements alternating in clockwise and counterclockwise directions. The reciprocating movement promotes increased resistance of the NiTi instrument to cyclical fatigue [6]. The WaveOne file has a different cross-sectional design along its entire active portion; the tip has a modified triangular cross-section, and the middle and neck portions of the working part of the instrument change to a neutral rake angle with a convex triangular transverse cross-section [7, 8]. The files have a reverse taper, variable helical angle and a non-active edge. It is used with 170^°^ counter clockwise rotation (direction of cutting) and 50^°^ clockwise rotations at a speed of 300 rpm. WaveOne is also available in different tip sizes and tapers 21/0.06 (small), 25/0.08 (primary) and 40/0.08 (large). This file is made of heat-treated NiTi *Memory Wire*, which also confers greater resistance to fatigue [9].

The OneShape system (Micro Méga, Besançon, France) is another single-file system that was developed for use in continuous rotation and is characterized by variable pitch, a noncutting safety tip and three variations of cross-sections along its active length: a changing triangular or modified triangular cross-section with 3 sharp cutting edges in the apical and middle part and an S-shaped design with 2 cutting edges near the shaft [8]. In severely curved canals, instrumentation is a critical step due to the difficulty of adjusting the instruments to the canal anatomy [2]. Therefore, an evaluation of the instrumentation files in these anatomies is needed.

Several methods have been proposed to identify the canal anatomy, such as radiographies [10], diaphonization [11], computed tomography (CT) [12], and more recently, micro-CT [13]. Micro-CT technology, allows observation of the root canals in two-dimensional (2D) and three-dimensional (3D) manner [14]. Moreover, the images allow for pre and postoperative evaluations, without the need to destroy the specimens [15].

This study aimed to evaluate and compare the morphological changes resulting from the instrumentation of severely curved root canals with these two single-file systems. The null hypothesis was that there would be no difference between the two systems in terms of 2D (area, perimeter, roundness, and minor and major diameters) and 3D (volume, surface area, transportation, and the Structure Model Index-SMI) parameters of the prepared root canal system.

## Materials and Methods

***Initial sample selection***

The study was reviewed and approved by the Research Ethics Committee of the University of Pernambuco (UPE); Pernambuco, Brazil and was performed in accordance to the Declaration of Helsinki (World Medical Association). A total of 307 maxillary molars were assessed with stereomicroscope under 4× magnification, according to the following criteria: intact roots, complete root formation and an intact pulp chamber. At this point 104 molars were selected. The teeth were disinfected in 0.1% thymol solution for 24 h and stored in saline. The palatal roots were sectioned with a carborundum disc to avoid radiographic superimposition. 

***Sample selection with digital radiographies***

The remaining 104 teeth were then radiographed in buccolingual and mesiodistal direction with a digital radiographic sensor (Digora, Soredex, Orion Corporation Ltd., Helsinki, Finland) to confirm the absence of pulp calcification, internal resorption, previous endodontic treatment and perforated roots. Thirty teeth were compatible with these characteristics and were excluded from the study. The curvature angles were measured in buccolingual and mesiodistal planes and were classified as severely curved (30^º^-50^º^), according to Schneider's method [16]. Finally, teeth with MB canals with a radii of curvature more than 10 mm [17] were excluded. A total of 38 canals remained in the sample at this point.

***Selection with computed tomography (CT)***

This step was used to select single root canals that extended from the pulp chamber to the root apex, which were classified as *Type I* according to Vertucci’s classification [18]. A cone-beam CT scanner (Soredex, Orion Corporation Ltd., Helsinki, Finland) was used with the following acquisition parameters: 90 kVp, 12.5 mA, voxel size of 85 µm, FOV of 6×4 cm and using the high-resolution EndoMode function. The sample contained 28 canals at this point. 

***Selection with micro-CT***

A custom jig for each tooth was created in order to repeat the same position for preoperative and postoperative scan. Images were obtained with a SkyScan 1174 v.2 (Bruker micro-CT, Kontich, Belgium) with the following acquisition parameters: 50 kV, 800 µA, 6-30 µm spatial resolution, 0.5 mm Al filter, 1^º^ rotation step, frame averaging of 3.5 and 180^º^ rotation. For reconstruction, the parameters used included: ring artifact correction of 5, 15% beam hardening correction and contrast limits from 0.015 to 0.095. This method was used to confirm a single canal (*Type I*) [18] and to standardize the initial canal volume. A total of 10 specimens were selected for the final sample.

A sample size calculation was performed based on previous article [19] and was considered an alpha of 5% and power of 80% or upper, which resulted in five samples per group (*n=*5). 

***Root canal preparation***

Endodontic access cavity was prepared and a glide path was created using a #10 and #15 K-files (Dentsply Maillefer, Ballaigues, Switzerland) [13] until the tip could be observed in the apical foramen. The procedures were performed with Dental Operating Microscopy (DF Vasconcellos S/A, São Paulo, SP, Brazil) under 8× magnification. The crowns of the teeth were cut off with a diamond blade in an ISOMET 1000 precision sectioning saw (Buehler, Lake Forest, IL, USA) until the root reached a total length of 17 mm. The working length (WL) was set as 1 mm short of the apical foramen. After numbering the samples, the teeth were randomly divided into 2 groups: WaveOne and OneShape groups. The procedures were performed by a single operator according to the manufacturer’s instructions and files were discarded after a single use in both groups. In the WaveOne group, a primary 25/0.08 file coupled to a gear reduction hand-piece (Sirona Dental Systems GmbH, Bensheim, Germany) powered by a torque-controlled motor (Silver; VDW GmbH, Munich, Germany) was used to prepare the canals in a reciprocating and slow in-and-out pecking motion. 

In the OneShape group, a 25/0.06 file was coupled to the same motor but was used in a continuous rotation mode at 350 rpm and 2.5 N.cm torque with pressure less in-and-out movements. After 3 in-and-out movements, the file was removed from the root canal, cleaned with a sponge and the canal was irrigated. The irrigation was carried out with 5 mL of 2.5% sodium hypochlorite and performed using a syringe and an open-end 30 G needle (NaviTip; Ultradent Products Inc, UT, USA) positioned 2 mm short of the WL. In both groups these steps were repeated until the file reached the WL. Cleaning after instrumentation consisted of irrigation with 5 mL 17% EDTA (Formula e Ação, São Paulo, SP, Brazil) followed by 5 mL 2.5% sodium hypochlorite (Formula e Ação) and 5 mL of deionized water (Formula e Ação). Canals were dried using paper points. 

***Micro-CT measurements and evaluation***

Images were reconstructed from the apex to the level of the cementoenamel junction (NRecon v1.6.4; Bruker), providing axial cross sections of the inner structure of the samples. For each tooth, an evaluation was conducted for the full canal length in approximately 794 slices per specimen (range of 636-918 slices). CTAn v1.11 software (CTAnalyser; Skyscan, Antwerp, Belgium) was used to obtain 2D morphological data (area, perimeter, roundness, major diameter and minor diameter). The round or more ribbon-shaped cross-sections, were expressed as round canals. This index varies from 0 (parallel plates) to 1 (perfect ball). The 2D evaluation was performed on the apical third of the tooth with 1-mm intervals, from the anatomic apex proceeding upward for 5 mm. 

The 3D morphological data analyses [volume, surface area, structure model index (SMI) and transportation] were obtained in the total root canal. Also, the canal transportation was analyzed in the cervical, middle and apical thirds (15 mm, 10 mm and 5 mm from the anatomic apex, respectively). The SMI involves measurement of a solid surface convexity. Their values vary from 0 to 4, and values 0, 3, and 4 correspond, respectively, to plane, cylinder and regular sphere. The 3D models of the root canals were obtained using an algorithm (Double Time Cubes in P3G format) and displayed in CTVol 2.1 software (CTAnalyser; Skyscan, Antwerp, Belgium). Detailed descriptions of the criteria used for the calculation of these parameters are provided by Versiani *et al.* [20]. Canal transportation was evaluated from the center of gravity obtained from the coordinates of the x, y and z axes according to the 3D Cartesian coordinate system. Two points were determined: P_1_=(x_1_, y_1_, z_1_) and P_2_=(x_2_, y_2_, z_2_), which corresponded to the central position of the same canal in the same cross-section before and after instrumentation. The distance between these two points was calculated using the following formula:$d=\sqrt[{2}]{{\left(\mathrm{x2}-\mathrm{x1}\right)}^{2}+(\mathrm{y2}-\mathrm{y1}{)}^{2}+{\left(\mathrm{z2}-\mathrm{z1}\right)}^{2}}$ ,where *d* is the distance between the two points. The assessment of the canal preparation was performed with micro-CT by another blinded examiner. 

***Statistical analysis***

The Kolmogorov–Smirnov test was used to determine the data distribution of each parameter. If the distribution was normal, a t-test for independent samples was used. The level of significance was set at 0.05.

## Results

The initial canal volume was similar between groups, with no statistical significant differences (*P*=0.58) (Table 1).

Regarding 3D parameters, the two file systems increased the surface area, volume and SMI after instrumentation of root canals and no significant differences between the groups were detected (surface area, *P*=0.637; volume, *P*=0.584; and SMI, *P*=0.370). No significant difference was observed between file systems in canal transportation in overall canal length (*P*=0.498), cervical (*P*=0.553), middle (*P*=0.498) and apical (*P P*=1.00) thirds of root canals (Tables 1 to 3). 

Regarding 2D parameters in apical third (Table 3), the canal area showed no statistically significant difference between the WaveOne and OneShape groups after instrumentation in all levels: 1 mm (*P*=0.809); 2 mm (*P*=0.068); 3 mm (*P*=0.052); 4 mm (*P*=0.053) and 5 mm (*P*=0.140). Regarding the perimeter, a significant difference in the apical third was found between the two groups in 3 mm (*P*=0.025) and 4 mm (*P*=0.039) areas. 

In terms of roundness, the OneShape group showed a significant difference between the original canal and the canal after instrumentation in the apical 3, 4 and 5 mm sections (Table 2). The change in roundness between groups was statistically significant for the 4-mm section (*P*=0.009).

**Table 1:** Three-dimensional analysis of MB canals after use of two different single-file systems

**Volume (mm** ^3^ **)**	**WaveOne**	**One Shape**
**Original **	1.93±0.85	1.66±0.64
**Mean (SD) of increase**	4.86±0.15*	4.05±0.77*
**Mean (SD) of increase (%)**	204.78±166.18	175.14±116.95
**Surface Area (mm** ^2^ **)**		
**Original **	19.97±6.00	18.42±3.80
**Mean (SD) of increase**	29.16±0.86*	25.28±3.21*
**Mean (SD) of increase (%) **	56.49±48.21	41.56±30.68
**SMI**		
**Original **	2.31±0.26	2.17±0.20
**Mean (SD) of increase**	2.74±0.14*	2.90±0.09*
**Mean (SD) of increase (%)**	19.39±8.40	34.91±12.05

* *Intragroup significant differences*

**Table 2:** Means (SD) of transportation in different canal areas

**Group**	**Total**	**Cervical third**	**Middle third**	**Apical third**
**WaveOne**	0.11 (0.03)	0.08 (0.03)	0.10 (0.06)	0.14 (0.03)
**One Shape**	0.12 (0.01)	0.09 (0.02)	0.12 (0.02)	0.14 (0.02)

Regarding diameter, the differences between the original and the prepared canals were mostly observed in the minor canal diameter but not in the major canal diameter (Table 2).

## Discussion

This study used extracted human teeth to better simulate the clinical conditions with regard to the morphological changes caused by the file systems used for instrumentation. The MB canals of upper molars were chosen given their high incidence of abrupt curvature in the apical third [21], which can adversely influence the canal preparation [22]. However, MB canals tend to vary considerably in their anatomy [23], which represents a challenge in terms of sample standardization [24]. The incidence of a second canal in the MB root of upper molars can vary between 18.6 to 100%, [23, 25], making the selection of single MB root canals of upper molars a critical point in research. Therefore, the rigorous standardization of specimens becomes vital in laboratory-based studies to ensure control of the experimental conditions of the study and only the variables

 of interest, such as the tested materials, remain in the analysis [26]. For this reason, great effort was placed into balancing the samples to minimize the influence of canal anatomy. 

Many researches used solely visual inspection of radiographies for anatomical classification and analysis of canal preparation [12, 14] and it was the second step in the sample selection for this study. Due to a large number of specimens to be evaluated, the use of radiographies in this methodology can be justified given its low cost and fast results. The standardization in this study was considered effective because it led to the exclusion of 63.4% of the initial specimens. However, digital radiography does not allow for the visualization of the canal curvature in all of its different planes and variations and of anatomical irregularities or convexities, which are common in root canals [27]. 

One of the advantages of cone-beam computed tomography (CBCT) is that it provides more detailed images of internal tooth anatomy than conventional periapical radiographs [28] and lead to more accuracy in the sample standardization. The selection of single canals using CBCT reduced the sample by 26.3%. 

**Table 3:** Two-dimensional morphological analysis of the apical third of MB canals of upper molars

	**Area (mm** ^2^ **)**		**Perimeter (mm)**		**Roundness (mm)**		**Major diameter (mm)**		**Minor diameter (mm)**	
	**WaveOne**	**One Shape**	**WaveOne**	**One Shape**	**WaveOne**	**One Shape**	**WaveOne**	**One Shape**	**WaveOne**	**One Shape**
**Level**										
**1 mm**										
**Original**	0.07 (0.07)	0.05 (0.01)	1.09 (0.56)	0.91 (0.19)	0.53 (0.23	0.47 (0.07)	0.42 (0.2)	0.36 (0.08)	0.21 (0.11)	0.17 (0.02)
	0.10 (0.09)	0.09 (0.01)*	1.11 ± 0.70	1.30 (0.19)*	0.73 (0.16	0.55 (0.15)	0.38 (0.26)	0.48 (0.09)	0.28 (0.14)	0.28 (0.03)*
	185.44 (373.97)	106.04 (36.78)	29.96 (119.81)	44.79 (13.99)	59.08 (76.24	7.99 (39.99)	18.39 (110.00)	33.41 (16.82)	78.41 (146.72)	67.55 (25.65)
**2 mm**										
**Original**	0.05 (0.02)	0.06 (0.05)	0.91 (0.26)	1.01 (0.29)	0.65 (0.20)	0.55 ( 0.12)	0.33 (0.11)	0.38 (0.11)	0.21 (0.02)	0.22 (0.07)
	0.14 (0.03)*	0.10 (0.03)*	1.39 (0.17)*	1.31 (0.30)	0.57 (0.23)	0.69 (0.20)	0.46 (0.03)	0.46 (0.14)	0.39 (0.05)*	0.32 (0.04)*
	212.67 (172.32)	102.95 (116.56)	65.39 (57.86)	36.97 (41.12)	-11.28 (19.04)	27.62 (39.13)	55.04 (60.03)	26.72 (41.00)	92.97 (19.18)	58.46 (50.99)
**3 mm**										
**Original**	0.08 (0.05)	0.09 (0.04)	1.24 (0.62)	1.21 (0.32)	0.56 (0.28)	0.56 (0.07)	0.48 ( 0.27)	0.45 (0.12)	0.25 (0.06)	0.27 (0.04)
	0.21 (0.06)*	0.14 (0.02)*	1.84 (0.27)†	1.46 (0.15)†	0.66 (0.23)	0.71 (0.11)*	0.64 (0.12)	0.50 (0.07)	0.48 (0.09)*	0.37 (0.04)*
	262.90 (236.15)	73.41 (66.19)	76.84 (75.35	25.82 (22.80)	28.87 (36.73)	25.79 (16.16)	67.69 (73.83)	12.90 (17.38)	96.26 (30.97)	43.09 (39.32)
**4 mm**										
**Original**	0.14 (0.10)	0.13 (0.09)	1.41 (0.68	1.60 (0.69)	0.62 (0.15)	0.43 ( 0.11)	0.52 (0.26)	0.63 (0.29)	0.33 ( 0.14)	0.27 (0.05)
	0.30 (0.09)*	0.19 (0.06)	2.16 (0.36)†	1.65 (0.29)†	0.69 (0.19)	0.80 (0.09)*	0.75 (0.17)	0.54 ( 0.29)	0.55 (0.09)*	0.45 (0.05)*
	264.59 (281.07)	78.31 (95.90)	80.06 (78.14	13.64 (32.79)	11.66 (15.34)†	96.55 (53.27)†	70.00 (68.30)	7.06 (26.78)	84.84 (76.68)	72.80 (35.78)
**5 mm**										
**Original**	0.16 (0.1)	0.15 (0.12)	1.53 (0.68)	1.58 (0.55)	0.61 (0.10)	0.48 (0.04)	0.56 (0.25)	0.61 (0.21)	0.34 (0.16)	0.28 (0.09)
	0.38 (0.10)*	0.26 (0.13)	2.40 (0.34)	1.89 (0.46)	0.60 (0.24)	0.78 (0.10)*	0.80 (0.13)	0.62 (0.15)	0.65 (0.09)*	0.57 (0.14)*
	355.87 (476.13)	105.75 (57.91)	90.49 (106.16)	22.18 (13.77)	5.23 (48.31)	64.18 (32.74)	74.69 (95.46)	5.18 (10.78)	131.14 (132.3)	109.56 (42.2)

∆: *mean increase (± standard deviation);*

∆ (%): *percentage mean increase (± standard deviation);*

*: *significant difference intragroup;*

† *: significant difference intergroup*

The micro-CT has been considered as the gold standard for laboratory studies in endodontics [11]. However, studies with upper molars showed no difference between the images obtained with micro-CT and CBCT in terms of canal detection [23]. In addition, CBCT images acquired with a voxel size less than 300 µm have been shown to be compatible with micro-CT images for the morphological study of hard tissues [29]. Nevertheless, the use of micro-CT in this study allowed for the visualization of anatomical complexities that were not visible with CBCT, leading to the exclusion of 64.2% of the specimens and a final sample of 10. Morphometric evaluation of root canals for sample selection was proposed in a previous study and authors also included specimen selected solely on basis of radiographies to strengthen the statistic power [30], which clearly shows the difficulty for using micro-CT as a methodology for sample selection. In the present study, final sample selection was established with micro-CT and statistic power was higher than 80%, calculated based on literature and recommend for researches [31]. 

The instrument shape can promote morphological changes during root canal preparation [22]. Although both systems in this study used single files with the same apical diameter, the taper of the files was different. According to the manufacturers, the One Shape file had a 0.06 taper along its active length, while the WaveOne file had a 0.08 taper in the initial 3 mm that decreases until D16. As the WaveOne shows greater taper, it can be inferred that this characteristic could be related to the significant increase in canal perimeter and roundness in the apical region observed in this group compared to the OneShape group. It can be deduced that both file systems exhibited similar cutting capacity because both groups showed significant increases in canal volume and surface area, although this difference was not significant between the groups. The files used in this study were made of NiTi, a metal that confers great flexibility to the instrument, thus favoring the maintenance of the canal curvature during preparation [32], which is a highly desirable property in complex anatomies such as severely curved canals. Moreover, the alloy of the WaveOne file is heat treated, leading to more flexibility and resistance to fatigue compared to traditional NiTi files [33].

In the apical third, less instrumentation of the original canal walls was performed. Even then, there were no statistically significant differences between groups, and no specimen showed root perforation. This finding is consistent with other studies that showed difficulty in cleaning the apical third of the canal [22, 34]. Therefore, the null hypothesis was rejected for roundness and perimeter in 2D parameters and was accepted for all other parameters analyzed in this research.

## Conclusion

The two tested file systems (WaveOne and One Shape) had similar shaping ability for severely curved MB canals of maxillary molars. Overall, both systems were able to maintain the original canal anatomy, producing minor changes in the canal curvature. This *in vitro *study showed that stereomicroscope, digital radiographies, cone-beam CT, and micro-CT can be suitable methods for obtaining uniform samples and minimizing potential anatomical bias. 
